# Correspondence Between Values of Vertical Loading Rate and Oxygen Consumption During Inclined Running

**DOI:** 10.1186/s40798-022-00491-2

**Published:** 2022-09-06

**Authors:** Marcel Lemire, Mathieu Falbriard, Kamiar Aminian, Eloïse Pavlik, Grégoire P. Millet, Frédéric Meyer

**Affiliations:** 1 Faculty of Science and Technology, University of Upper Alsace, Mulhouse, France; 2 Institute of Sport Sciences, University of Lausanne, Lausanne, Switzerland; 3 Digital Signal Processing Group, Department of Informatics, University of Oslo, Oslo, Norway; 4 IRIMAS, University of Haute-Alsace, Mulhouse, France; 5 Laboratory of Movement Analysis and Measurement, EPFL, Lausanne, Switzerland

**Keywords:** Biomechanics, Energy cost of running, Vertical loading rate, Inclined treadmill

## Abstract

**Purpose:**

The aim of this study was to provide a theoretical model to predict the vertical loading rate (VLR) at different slopes and speeds during incline running.

**Methods:**

Twenty-nine healthy subjects running at least once a week performed in a randomized order 4-min running trials on an instrumented treadmill at various speeds (8, 10, 12, and 14 km h^−1^) and slopes (− 20%, − 10%, − 5%, 0%, + 5%, + 10%, + 15%, + 20%). Heart rate, gas exchanges and ground reaction forces were recorded. The VLR was then calculated as the slope of the vertical force between 20 and 80% of the duration from initial foot contact to the impact peak.

**Results:**

There was no difference in VLR between the four different uphill conditions at given running speeds, but it was reduced by 27% at 5% slope and by 54% at 10% slope for the same metabolic demand (similar ${\dot{\text{V}}\text{O}}_{{2}}$), when compared to level running. The average VLR measured at maximal aerobic intensity during level running would be decreased by 52.7% at + 5%, by 63.0% at + 10%, and by 73.3% at + 15% slope. Moreover, VLR was dependent on the slope in downhill conditions.

**Conclusion:**

This study highlights the possibility to use uphill running to minimize rate of mechanical load (i.e., osteoarticular load) from foot impact on the ground and as a time-efficient exercise routine (i.e., same energy expenditure than in level running in less time).

## Key Points

- The vertical loading rate (VLR) remains similar in a wide range (5–20%) of uphill running.
- VLR is reduced by 53% at 5% and by 63% at 10% slope for the same metabolic demand (similar ${\dot{\text{V}}\text{O}}_{{2}}$), when compared to level running.
- A model predicting VLR from ${\dot{\text{V}}\text{O}}_{{2}}$ considering running velocity and terrain inclination is provided.

## Introduction

Running on hills modifies the running technique as well as the demands on the musculoskeletal system. Indeed, the energy cost of running (ECR) increases proportionally to the positive slope, whereas when running downhill, it decreases until a slope of − 20%, beyond which ECR increases again [1]. Biomechanics also change when running on a slope, compared to level running. In uphill running, the stride frequency is higher with a shorter swing phase duration, and the muscle activation is greater [2]. The propulsive force is also higher, which puts more strain on the posterior chain including the plantar flexor muscles, Achilles tendon and plantar fascia (i.e., concentric muscle action) [3], but the ankle joint moment is lower at an equivalent speed. Paradoxically, this would act to reduce the strain on plantar flexor muscles and Achilles’ tendon [4]. In downhill, the braking phases (i.e., peak forces) are higher than on level, inducing a higher stress on the heel, iliotibial band, and patella (i.e., eccentric muscle action). The swing phase is prolonged and the vertical displacement of the center of mass shows an increased amplitude [5]. The influence of the slope on the running biomechanics therefore has consequences for performance and injury prevention. Indeed, overuse injuries are largely caused by the application of repeated forces when the foot hit the ground [6]. The vertical loading rate (VLR), defined as the rate of change of the force application after the impact, has been used as the best indicator to describe ground impact [7]. Additionally, common pathologies in runners (i.e., patellofemoral syndrome, tibial stress fracture, and plantar fasciitis) have been linked to VLR [8], although recent studies reported no relationship between VLR and injury risk in level running [9, 10], contrary to lower step rate [11] or lower duty factor [9]. However, downhill running elicits very greater VLR values than in level running and a lower step rate at the same speed as in uphill running [12]. The loading rate appears to increase in downhill (− 6% slope) versus level running, but this increase was not significant on a − 9% slope [13] in a small group (*N* = 6). In another study, the normal and parallel ground reaction forces (expressed in N kg^−1^) were 2.5 times greater during the first half of stance phase during downhill than uphill running (± 20% slope at 12 km h^−1^) [14]. A visual inspection of the presented impact forces shows that the VLR in downhill is approximately twice the VLR in uphill. Using uphill running as a strategy to reduce VLR while keeping a equivalent metabolic stress could be interesting to prevent injuries. Indeed, it has been shown that an interval training program in uphill running was as effective as level running to improve maximal oxygen consumption (${\dot{\text{V}}\text{O}}_{{{\text{2max}}}}$) and percentage of ${\dot{\text{V}}\text{O}}_{{{\text{2max}}}}$ at lactic threshold [15]. Thus, it would be beneficial to know if an uphill running workout at equal energy cost to a level running workout has lower VLR. Indeed, VLR is influenced by the slope and the running speed with an increase in the loading rate in downhill running at high speeds [1]. Uphill running may induce a lower VLR for a given rate of energy expenditure level than level running and providing a theorical model of this relationship would be helpful for the communities of scientists and coaches.

Therefore, the purpose of this study was to provide a model of VLR conversion for the minimization of VLR at a given cardiovascular stress between level, downhill and uphill running at different speeds. We tested the hypothesis that the greater the positive slopes, the greater the decrease in VLR for a given quantified oxygen consumption, and actually a VLR conversion model would be established between metabolic load and VLR under different running speed and slope conditions, with lower VLR in favor of uphill conditions.

## Methods

### Experimental Design

Twenty-nine runners (19 males, 10 females) of varying levels of aerobic fitness (age: 34 ± 10 [mean ± SD] years; height: 1.74 ± 0.09 m; body mass: 68.3 ± 12.2 kg; ${\dot{\text{V}}\text{O}}_{{{\text{2max}}}}$: 56.6 ± 8.9 ml min^−1^ kg^−1^) performed four sessions: a level running incremental test followed by three sessions with running bouts of 4 min each, at constant speeds (8, 10, 12 or 14 km h^−1^) and slopes (− 20%, − 10%, − 5%, 0, + 5%, + 10%, + 15% or + 20%) with 1 week resting periods in-between the three sessions. Seven to eight running bouts were performed in each of the three sessions in a randomized sequence to perform a total of 25 conditions, but with a similar exercise load between sessions. Each running bout was followed by 2 min of rest for intensities lower than the first ventilatory threshold, and 5 min of rest for higher intensities. Conditions where the participants were not able to reach a steady state were aborted. All running sessions were performed on an instrumented treadmill (T-170-FMT, Arsalis, Belgium) in Lausanne (Switzerland). Participants were recruited in the local population and represented a wide range of aerobic fitness, running between one and five times a week. They were all familiar with treadmill running but were not trail specialists. All subjects signed an informed consent. The protocol was approved by the local ethical committee (CER-VD 2015–00,006) and conducted according to the declaration of Helsinki.

### Physiological Parameters

Gas exchanges were measured breath by breath (Quark CPET, Cosmed, Rome, Italy) and volume and gas calibrations were checked before each session. Heart rate was continuously measured (Polar Electro, Kempele, Finland). Blood lactate concentration was assessed from finger blood samples (Lactate Scout+, EKF Diagnostics, Leipzig, Germany) before the maximal incremental running test and after 1 and 3 min of recovery. For the constant-speed running bouts sessions, the blood samples were collected after 3 min of recovery.

### Maximal Incremental Level Running Test

All participants performed an incremental running test until exhaustion. The first stage began at 8 km h^−1^ for 4 min and then increased by 1 km h^−1^ every min.

### Energy Cost of Running Trials

As indicators of running economy, ECR was normalized by body weight (BW) and averaged over 30 s for each running condition by dividing the mean ${\dot{\text{V}}\text{O}}_{{2}}$ by the velocity and multiplied by the energy equivalent of O_2_ estimated by respiratory exchange ratio to be expressed in J kg^−1^ m^−1^ [16].

### Biomechanical Parameters

Ground reaction forces in the vertical (VGRF), forward, and lateral components were continuously recorded with an instrumented single-belt treadmill (T170—FMT-MED; Arsalis, Belgium) at a sampling rate of 1000 Hz. The calculation of the various biomechanical parameters has been described previously [16, 17]. The VLR was calculated as the average VGRF slope between 20 and 80% of the point where the VGRF slope is less than 15 BW s^−1^ [18]. The VLR was then averaged over the 30 s of recordings corresponding to 70–90 consecutive steps.

### Statistical Analysis

Due to the lack of several conditions, linear mixed models (LMM) were fitted with a random intercept effect for subjects, Fixed effects were analyzed for treadmill slopes and running speeds as ordinal variables (Jamovi 1.2, Sydney; Australia). Significance of fixed effects was evaluated with an analysis of variance (Restricted Maximum Likelihood—REML—estimation). As VLR, ECR and ${\dot{\text{V}}\text{O}}_{{2}}$ were used as dependent variables, Bonferroni’s correction was applied on the alpha level to account for repeated univariate testing. After fitting the LMM, the residuals were checked for normality using the Kolmogorov Smirnov test and post-hoc comparisons were performed within groups with Bonferroni’s multiplicity correction.

Pearson’s product moment correlation coefficients (*r*) were used to assess the intensity of the relations between variables using Statistica (13.5, Tulsa, Oklahoma, USA). For all these analyses, *p* < 0.05 was considered statistically significant. All data are expressed as mean ± standard deviation (SD).

## Results

The VLR increased when running speed increased (*p* < 0.001; *F* = 87.9) and when slope decreased (*p* < 0.001; *F* = 93.9). The ECR significantly increased when running speed decreased (*p* = 0.009; *F* = 4.2) and when slope increased (*p* < 0.001; *F* = 1453.1). Finally, the ${\dot{\text{V}}\text{O}}_{{2}}$ increased when running speed increased (*p* < 0.001, *F* = 459.0) and when slope increased (*p* < 0.001; *F* = 804.0). All the post-hoc tests are presented in Table 1. All residuals of the LMM were normally distributed (all *p* > 0.05).

**Table 1:** Effect of treadmill speed and slope on vertical load rate and energy cost of running

Slope (%)		Treadmill Speed (km h^−1^)			
		8	10	12	14
+ 20	VLR (N s^−1^ kg^−1^)	187.5 ± 22.0^ac^			
	ECR (J kg^−1^ m^−1^)	9.9 ± 0.6	–	–	–
	${\dot{\text{V}}\text{O}}_{{2}}$ (ml kg^−1^ min^−1^)	62.7 ± 4.7			
+ 15	VLR (N s^−1^ kg^−1^)	184.2 ± 44.8^aα^	217.6 ± 43.1^aα^		
	ECR (J kg^−1^ m^−1^)	7.7 ± 1.2^α^	8.1 ± 0.6^α^	–	–
	${\dot{\text{V}}\text{O}}_{{2}}$ (ml kg^−1^ min^−1^)	52.8 ± 6	65.1 ± 3.7		
+ 10	VLR (N s^−1^ kg^−1^)	181.2 ± 54.3^aα^	195.6 ± 60.8^abαβ^	238.1 ± 67.6^aγ^	
	ECR (J kg^−1^ m^−1^)	6.7 ± 0.6^α^	6.5 ± 0.5^α^	6.4 ± 0.3^α^	–
	${\dot{\text{V}}\text{O}}_{{2}}$ (ml kg^−1^ min^−1^)	47.0 ± 3.6	54.6 ± 3.7	63.2 ± 2.9	
+ 5	VLR (N s^−1^ kg^−1^)	199.7 ± 80.2^aα^	246.9 ± 88.6^abβ^	308.0 ± 104.6^aγ^	318.0 ± 120.9^aγ^
	ECR (J kg^−1^ m^−1^)	5.1 ± 0.5^α^	4.9 ± 0.6^α^	4.9 ± 0.7^α^	5.0 ± 0.5^α^
	${\dot{\text{V}}\text{O}}_{{2}}$ (ml kg^−1^ min^−1^)	38.3 ± 3.3	43.2 ± 4.0	50.1 ± 5.3	58.3 ± 3.5
0	VLR (N s^−1^ kg^−1^)	210.3 ± 91.4^abα^	279.5 ± 134.4^abαβ^	337.0 ± 149.0^aβ^	420.3 ± 184.6^abγ^
	ECR (J kg^−1^ m^−1^)	4.0 ± 0.5^α^	3.9 ± 0.4^α^	3.9 ± 0.6^α^	4.0 ± 0.3^α^
	${\dot{\text{V}}\text{O}}_{{2}}$ (ml kg^−1^ min^−1^)	31.4 ± 3.2	37.2 ± 3.0^a^	43.0 ± 4.7	49.0 ± 3.3
− 5	VLR (N s^−1^ kg^−1^)	269.6 ± 117.7^bcα^	335.8 ± 133.7^aβ^	435.8 ± 164.7^abγ^	499.6 ± 167.1^bcδ^
	ECR (J kg^−1^ m^−1^)	3.2 ± 0.4^α^	3.0 ± 0.4^αβ^	2.9 ± 0.5^β^	3.0 ± 0.4^αβ^
	${\dot{\text{V}}\text{O}}_{{2}}$ (ml kg^−1^ min^−1^)	26.2 ± 2.9	29.8 ± 2.9	33.8 ± 4.4	39.4 ± 3.4
− 10	VLR (N s^−1^ kg^−1^)	340.6 ± 147.4^dα^	409.6 ± 145.3^cβ^	507.1 ± 168.7^bγ^	597.0 ± 181.9^cδ^
	ECR (J kg^−1^ m^−1^)	2.8 ± 0.6^α^	2.6 ± 0.5^aβ^	2.5 ± 0.4^aβ^	2.6 ± 0.4^β^
	${\dot{\text{V}}\text{O}}_{{2}}$ (ml kg^−1^ min^−1^)	23.8 ± 3.8	26.8 ± 4.0	30.2 ± 3.9^a^	34.6 ± 4.1
− 20	VLR (N s^−1^ kg^−1^)		633.3 ± 140.3^dα^	756.8 ± 165.6^cβ^	884.6 ± 166.3^dβ^
	ECR (J kg^−1^ m^−1^)	–	2.2 ± 0.4^aα^	2.2 ± 0.5^aβ^	2.1 ± 0.5^γ^
	${\dot{\text{V}}\text{O}}_{{2}}$(ml kg^−1^ min^−1^)		23.9 ± 3.6^α^	27.1 ± 4.9^aαβ^	29.3 ± 6.3^β^

Values are means ± SD, Vertical load rate (VLR) normalized by bodyweight, energy cost of running (ECR) and oxygen consumption (${\dot{\text{V}}\text{O}}_{{2}}$). Superscript letters represent post-hoc pairwise comparisons with the same letters being not statistically different. Comparison between slopes at the same speed are represented using a < b < c < d (mean increasing order), while comparison between speeds at same slope use α < β < γ < δ at *p* < 0.05.

The equation of linear regression for VLR was:

1 $${\text{VLR }} = { }24.6891 \cdot {\text{speed }} + { }16.1520 \cdot {\text{slope }} - { }2.7037 \cdot {\text{speed}} \cdot {\text{slope}} + { }73.2095{ }$$

With a correlation coefficient *r*^2^ = 0.96 and where VLR is expressed in N s^−1^ kg^−1^, speed in km h^−1^ and slope in %.

And the equation of the linear regression for ${\dot{\text{V}}\text{O}}_{{2}}$ was:

2 $${\dot{\text{V}}\text{O}}_{{2 }} = { }2.384 \cdot {\text{speed }} + { }1.2594 \cdot {\text{slope }} - { }0.0015 \cdot {\text{speed}} \cdot {\text{slope }} + { }16.9999$$

With a correlation coefficient *r*^2^ = 0.90 and where ${\dot{\text{V}}\text{O}}_{{2}}$ is expressed in ml kg^−1^ min^−1^, speed in km h^−1^ and slope in %.

**Fig. 1:**
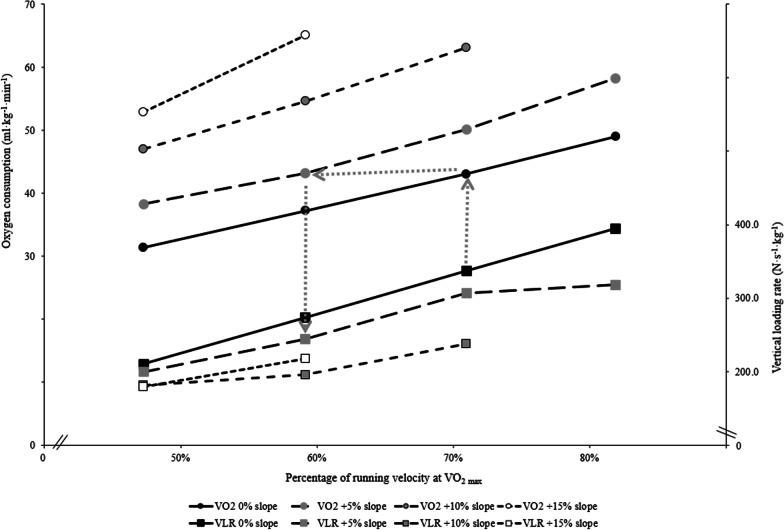
Relationships beween oxygen consumption, vertical loading rate (VLR) and percentage of running velocity at ${\dot{\text{V}}\text{O}}_{{\text{2 max}}}$. The grey dotted arrows illustrate an example: if one runs on the flat at 70% of velocity at ${\dot{\text{V}}\text{O}}_{{\text{2 max}}}$ corresponding approximately here to 12 km h^−1^, then VLR is 337 N s^−1^ kg^−1^ (black solid line with black squares). The oxygen consumption is 43 ml kg^−1^ min^−1^ (black solid line with black rounds). Now, if one runs on a uphill at + 5% slope, for the same metabolic demand (similar ${\dot{\text{V}}\text{O}}_{{2}}$), then VLR will be reduced to 247 N s^−1^ kg^−1^ (black dashed line with gray squares). SD values have been omitted for clarity

At similar ${\dot{\text{V}}\text{O}}_{{2}}$, the average VLR measured during level running was decreased by 52.7% at + 5%, by 63.0% at + 10%, and by 73.3% at + 15% slope. For instance, from a practical standpoint, to achieve a given ${\dot{\text{V}}\text{O}}_{{2}}$ on a + 15% slope instead of a + 5% slope, running speed would be reduced by one-third and VLR would be reduced by ~ 40%. Using the graphical readout (Fig. 1), we can quantify the reduction in VLR by increasing the positive slope while maintaining a similar metabolic expenditure to flat running. Running at a speed corresponding to approximately 80% of one's flat velocity at ${\dot{\text{V}}\text{O}}_{{{\text{2max}}}}$ ($\text{v}{\dot{\text{V}}O}_{{{\text{2max}}}}$) on a + 5% slope, or 60% $\text{v}{\dot{\text{V}}O}_{{{\text{2max}}}}$ on a + 10% slope, or 50% $\text{v}{\dot{\text{V}}O}_{{{\text{2max}}}}$ on a + 15% slope was equivalent to a stimulus of flat $\text{v}{\dot{\text{V}}O}_{{{\text{2max}}}}$ stimulus (Fig. 1). Using the equations (Eqs. 1 and 2), VLR is reduced by 59% in uphill running (10% slope) at 6.75 km h^−1^ compared to level running at 12 km h^−1^, (218 vs. 369 N s^−1^ kg^−1^, respectively) for the same metabolic demand.

There was no correlation between ECR and VLR in any uphill running condition. A significant correlation appeared only between ECR and VLR at 10 km h^−1^ and 12 km h^−1^ running speed on − 10% slope (*r* = 0.59 and 0.64, respectively, both *p* < 0.05; Table 2).

**Table 2:** Relationship between vertical load rate and energy cost of running as function of treadmill slope and speed

Slope (%)	Treadmill Speed (km h^−1^)											
	8			10			12			14		
	*r*	*p*	*N*	*r*	*p*	*N*	*r*	*p*	*N*	*r*	*p*	*N*
+ 20	0.62	1.000	6		–			–			–	
+ 15	0.01	1.000	11	0.57	1.000	5		–			–	
+ 10	0.39	1.000	25	0.04	1.000	20	0.08	1.000	8		–	
+ 5	0.23	1.000	25	0.09	1.000	21	0.49	1.000	16	0.51	1.000	11
0	0.42	1.000	23	0.27	1.000	29	0.38	0.975	29	0.23	1.000	27
− 5	0.31	1.000	24	0.23	1.000	25	0.56	0.200	21	0.39	1.000	24
− 10	0.46	0.450	26	0.59	0.025^†^	26	0.64	0.025^†^	24	0.16	1.000	25
− 20				0.19	1.000	20	0.27	1.000	19	0.08	1.000	11

*r* is presented to show positive and negative correlations

^†^*p* < 0.05

## Discussion

The present study reports VLR, ${\dot{\text{V}}\text{O}}_{{2}}$ and ECR at multiple inclines and speeds in a large group of runners of diverse training levels and show that uphill running elicits higher metabolic load compared to level running while minimizing ground impacts.

The present results confirm that VLR decreases by increasing positive slope but extend the previous work by Gottschall et al. [19] by combining physiological responses to running biomechanics associated with treadmill speed and slope change in a large group of athletes. The results also provide new insights into VLR with respect to ECR in downhill running, which elicits specific cardiorespiratory responses (i.e., lower metabolic demand, more superficial ventilation pattern, exacerbated heart rate) [16, 20] relative to level running, whereas it increases VLR, presumably due to increased braking forces to absorb the ground impact itself associated with eccentric muscle actions. The muscles involved during extension forcibly lengthen under the potential effect of gravity to limit the drop-down of the center of mass. This eccentric muscle’s action requires less energy than a concentric muscle contraction, and part of the potential energy from the vertical oscillation of the center of mass is either mostly dissipated as wasted heat or stored in the muscle–tendon units during the braking phase before its recoil in the pushing phase. The stretch–shortening cycle is mainly involved during downhill running. This mechanism could partially explain the correlations found between ECR and VLR in two negative slopes (Table 2), but the reason why only two conditions provide significant differences remains unclear. Furthermore, given the large number of subjects, one can attest that uphill running at a similar ${\dot{\text{V}}\text{O}}_{{2}}$ to level running elicits a lower VLR, as illustrated in Fig. 1. Therefore, these results provide a metabolic conversion from level to uphill running intensity (i.e., ${\dot{\text{V}}\text{O}}_{{2}}$), while highlighting the loading rate involved. Even if injuries are caused by multiple factors as training overload and non-biomechanical factor in isolation, one may assume that these loading rates are partly responsible for musculoskeletal stress. Our results also specify the role of uphill running as a time-efficient exercise routine, whereby one can burn in less time the same number of calories as during a bout of level running.

Nevertheless, downhill conditions associated with low ECR cannot be substituted by uphill conditions, whatever the slope and/or the running speed. Indeed, downhill running generates high VLRs, with no ${\dot{\text{V}}\text{O}}_{{2}}$ correspondence in the other modalities (flat and uphill), regardless of speed. High VLRs can also be a goal of training, in the absence of injury, in this case to generate significant mechanical/neuromuscular stimuli.

Vertical load rate represents how quickly the impact force is applied (i.e., a steeper slope means a more rapid collision), potentially influencing training tolerance. To reduce VLR and the well-known effects of eccentric muscle action, the increase in mechanical solicitation has to be progressive, by modulating duration, speed and slope of the training sessions. Therefore, we do recommend starting the physical preparation with uphill intervals at high slopes and gradually using fewer steep slopes.

## Conclusions

This study highlights the possibility to use uphill running to minimize ground impacts and increase energy expenditure in lesser time. This strategy is likely an effective option to provide an alternative to the high VLR in downhill running, and the conversion models provided are relevant and useful to achieve this goal. Further studies are needed to show how this strategy would allow the body to progressively create adaptations and become more tolerant to impact. In addition, our results allow to estimate similar ECR during uphill than during level running by modulating either the running speed or the inclination of the slope.

## Data Availability

Due to ethical restrictions, the datasets generated for this study are available on request to the corresponding author.

## Abbreviations

ECR: Energy cost of running in J kg^−1^ m^−1^
VLR: Vertical loading rate in N s^−1^ kg^−1^
${\dot{\text{V}}\text{O}}_{{2}}$: Oxygen consumption in mlO_2_ kg^−1^ min^−1^
${\dot{\text{V}}\text{O}}_{{{\text{2max}}}}$: Maximal oxygen consumption in mlO_2_ kg^−1^ min^−1^
$\text{v}{\dot{\text{V}}O}_{{{\text{2max}}}}$: Running velocity at maximal oxygen consumption in km h^−1^
